# Targeting *Drosophila* Sas6 to mitochondria reveals its high affinity for Gorab

**DOI:** 10.1242/bio.059545

**Published:** 2022-11-18

**Authors:** Levente Kovacs, Agnieszka Fatalska, David M. Glover

**Affiliations:** Division of Biology and Biological Engineering, California Institute of Technology, Pasadena, CA 91125, USA

**Keywords:** Centriole, *Drosophila*, Golgi, Mitochondrial targeting

## Abstract

The ability to relocalize proteins to defined subcellular locations presents a powerful tool to examine protein-protein interactions that can overcome a tendency of non-targeted exogenous proteins to form inaccessible aggregates. Here, we show that a 24-amino-acid sequence from the *Drosophila* proapoptotic protein Hid's tail anchor (HTA) domain can target exogenous proteins to the mitochondria in *Drosophila* cells. We use this HTA tag to target the *Drosophila* centriole cartwheel protein Sas6 to the mitochondria, and show that both exogenous and endogenous Gorab can be co-recruited from the Golgi to the new mitochondrial site. This accords with our previous observation that monomeric *Drosophila* Gorab binds Sas6 to become centriole associated with a 50-fold greater affinity than dimeric Gorab binds Rab6 to become localized at the Golgi. Strikingly, *Drosophila* Sas6 can bind both *Drosophila* Gorab and its human GORAB ortholog, whereas human SAS6 is unable to bind either GORAB or Gorab. We discuss these findings in relation to the evolutionary conservation of Gorab and the divergence of Sas6, possibly reflecting known differences in persistence of the cartwheel in the centriole duplication cycle of fly and human cells.

## Teaser

**Summary:** A method for relocalizing proteins from the Golgi or centrosomes to the mitochondria in *Drosophila* cells is used to investigate the affinities of these proteins for each other.

## INTRODUCTION

The centriole is the 9-fold symmetrical structure at the core of the centrosome, the major organizing center for cytoplasmic microtubules. It is found closely associated with the Golgi apparatus, the major hub for vesicle trafficking. Indeed, microtubule nucleating proteins are known to be associated with and shared between the outer parts of both centrosomes and Golgi (Rivero et al., 2009; Rios, 2014). Surprisingly, however, we recently described a physical interaction between Sas6, which forms the 9-fold symmetrical cartwheel at the inner core of the procentriole, and a trans-Golgi associated protein, Gorab, which is essential for centriole duplication in *Drosophila* (Kovacs et al., 2018). We found that the Gorab protein is organized differently at the Golgi and at the centriole: at the trans-Golgi it homodimerizes through its coiled-coil domain, which binds to Rab6; at the centriole, part of the Gorab monomer's coiled-coil domain undergoes an antiparallel interaction with part of the coiled-coil of dimeric Sas6 to form a heterotrimeric complex. Our previous study indicated that although Rab6 can interact with Gorab *in vitro* just as it does at the Golgi, Rab6 cannot form a complex with Gorab *in vitro* if Gorab is already in complex with its centriolar interacting partner, Sas6. This is the consequence of the stronger interaction between Sas6 and Gorab relative to Gorab and Rab6 (Fatalska et al., 2021). Accordingly, overexpression of Sas6 in *Drosophila* led to the reduction of Gorab signal in the Golgi (fig. 4C in Fatalska et al., 2021). Together, these observations led us to hypothesize that because Sas6 binds Gorab with high affinity, elevated expression of Sas6 at other cellular locations should provide a general means for capturing Gorab and depleting it from the Golgi. To test this hypothesis, we wished to relocalize Sas6 to an ectopic site and determine whether Gorab would be directed from the Golgi to this site.

**Fig. 1.**
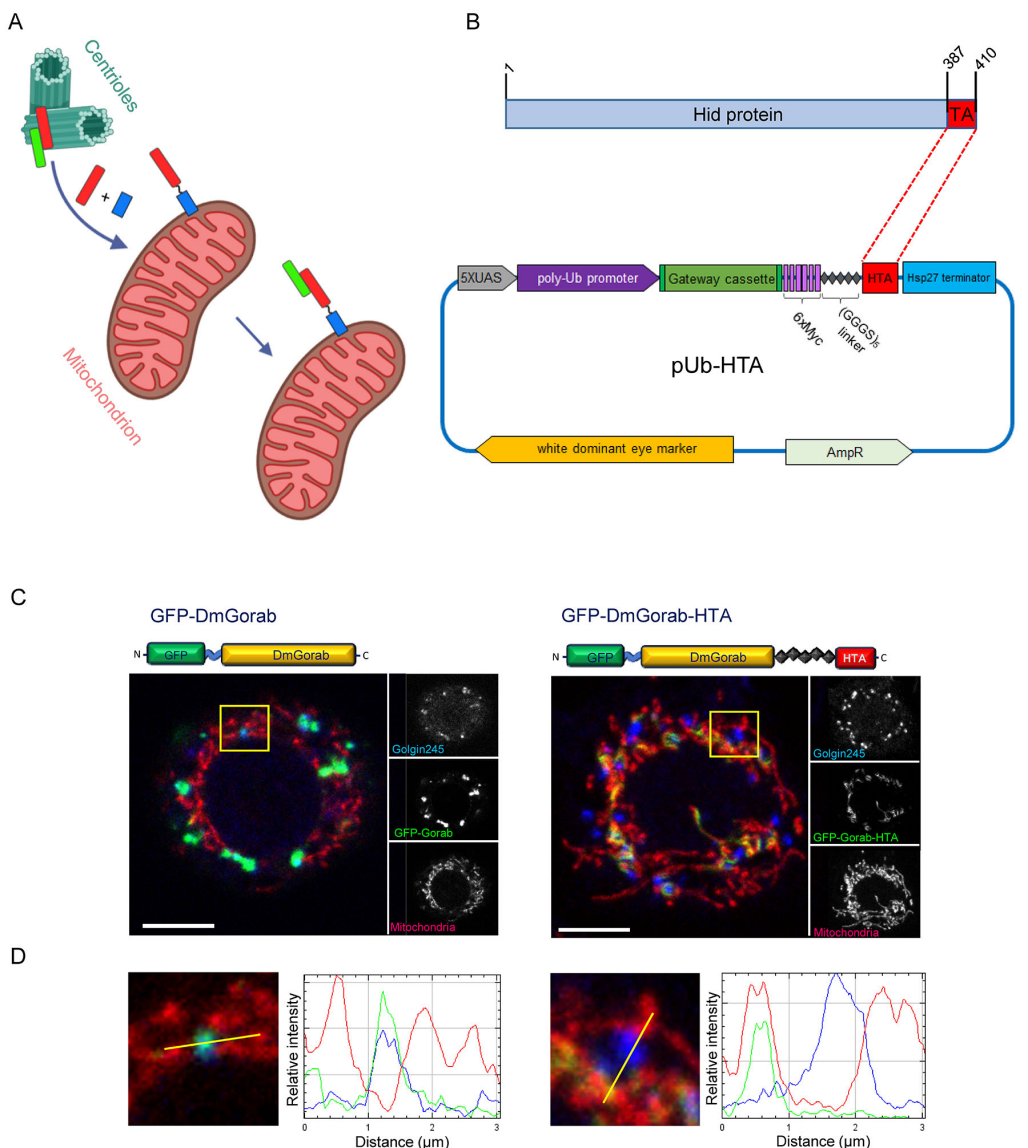
**Proteins tagged with the tail anchor of the *Drosophila* mitochondrial protein Hid localize to the mitochondria.** (A) Scheme representing the concept of mitochondrial relocalization. Protein A (red) and Protein B (green) interact at a given subcellular compartment, such as the centriole as shown here. If exogenous Protein A is tagged with a mitochondrial localization signal (blue), it will localize to the mitochondria. Subsequently, the endogenous binding partner of the relocalized protein, Protein B, will also localize to the mitochondria. (B) Map of the expression vector generated for mitochondrial targeting using Hid tail anchor (HTA). (C) Localization of GFP-Gorab with or without a C-terminal HTA tag in S2 cells transiently transfected with the indicated constructs. Cells were stained with MitoTracker Red and anti-Golgin245 antibody to reveal mitochondria and trans-Golgi, respectively. Scale bars: 5 μm. (D) Insets from C at 4× magnification. Yellow lines indicate pixel scans for the RGB fluorescence intensity profile plots shown on the right. *n*=30 cells analyzed per construct transfected. Experiment repeated three times with the same result.

We chose to use the mitochondrion as an ectopic targeting site because of the ease by which immunofluorescence could be used to track interacting partner proteins or protein complexes to this new compartment (Fig. 1A). Previous mitochondrial relocalization experiments in *Drosophila* have typically used fusions with large mitochondrial resident proteins to direct proteins inside the mitochondrial lumen (Andreazza et al., 2019) or with the 51-residue-long mitochondrial targeting sequence of human FIS1 that targets the outer mitochondrial membrane (McWilliams et al., 2016; Lee et al., 2018). We wished to avoid the use of human targeting sequences and so searched for possible alternatives within the *Drosophila* proteome. The proapoptotic protein Hid requires localization to the mitochondria for its function (Haining et al., 1999; Abdelwahid et al., 2007; Sandu et al., 2010). Hid is targeted to the mitochondrion by its C-terminal-most 24 residues, which form a hydrophobic tail anchor to the outer mitochondrial membrane (Haining et al., 1999; Sandu et al., 2010; Morishita et al., 2013). Here, we demonstrate that a 24-amino-acid sequence corresponding to the Hid tail anchor (HTA) domain can target exogenous proteins to the mitochondria in *Drosophila* cells. When *Drosophila* Sas6 is targeted to the mitochondria in this way, it is able to recruit both exogenous and endogenous Gorab to the same site.

## RESULTS

### The tail anchor of the *Drosophila* mitochondrial protein Hid targets recombinant proteins to the mitochondria

To develop a vector suitable for mitochondrial relocalization experiments, we incorporated the HTA, consisting of the terminal 24 residues, into a Gateway-compatible expression vector with a poly-ubiquitin (pUb) promoter to generate the pUb-HTA vector (Fig. 1B). The Gateway system provides an efficient and fast system for subcloning open reading frames between a variety of vectors (Akbari et al., 2009), and the pUb promoter is widely expressed in many cell types. As only the N-terminal 14 residues of Hid are required for induction of apoptosis, the tagging of non-apoptotic proteins with the C-terminal HTA will not induce apoptosis.

To test the efficiency of the mitochondrial relocalization, we first decided to use it to relocalize Gorab. Normally, Gorab is confined to the centrioles and Golgi in *Drosophila* cells (Kovacs et al., 2018; Fatalska et al., 2021), and neither of these structures overlaps with mitochondria in S2 cells. We found that, following transient transfection of GFP-Gorab without an HTA tag, the excess of Gorab was localized to the Golgi (Fig. 1C,D, left panel). However, in S2 cells transiently transfected with GFP-Gorab tagged with HTA, the GFP signal was shifted from the Golgi to the mitochondria (Fig. 1C,D, right panel). We noticed that although the GFP-Gorab-HTA signals overlapped with MitoTracker Red signal, the GFP signal was not evenly spread along the entire mitochondrial system. A similar nonuniform distribution has been observed for proteins targeted to the mitochondria with the C-terminal 51 residues of human FIS1 (Lee et al., 2018). These observations suggest that integration of the tail anchors may occur in preferred domains of the mitochondrial membrane. Nevertheless, the shift of GFP-Gorab away from the Golgi to the mitochondria in the presence of HTA tag points to the efficiency of this relocalization method.

### Mitochondrially targeted recombinant proteins can recruit their exogenous binding partners

**Fig. 2.**
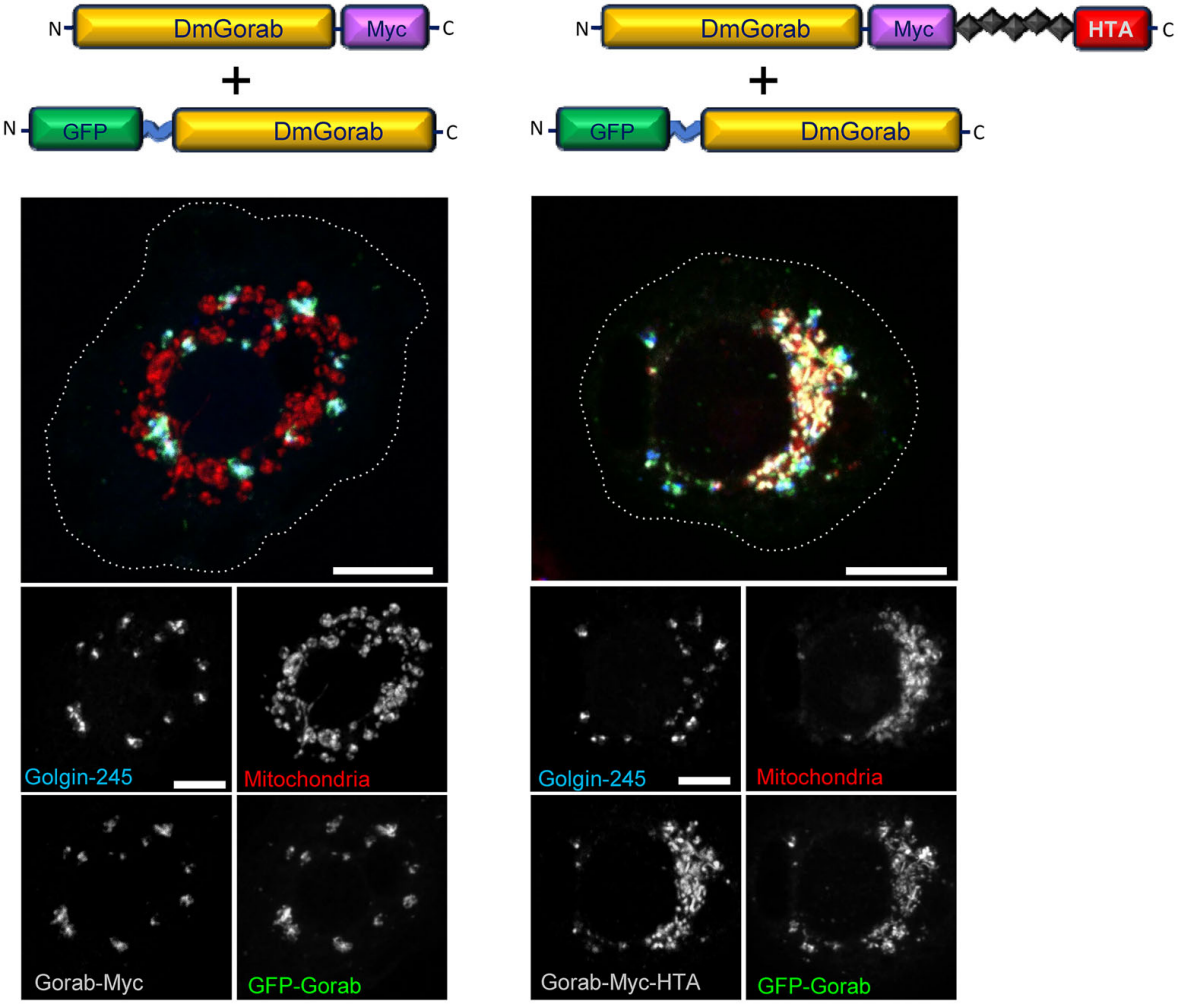
**Homodimerization of the mitochondrially targeted Gorab.** Constructs expressing Gorab-6xMyc without (left) or with (right) an HTA tag were co-transfected together with a construct expressing GFP-tagged Gorab into S2 cells. Cells were stained with MitoTracker Red, anti-Golgin245 antibody and anti-Myc antibody to reveal mitochondria, trans-Golgi and Gorab-Myc, respectively. Scale bars: 5 μm. *n*=30 cells analyzed per construct transfected. Experiment repeated three times with the same result.

We next wished to determine that the HTA tag providing the ectopic targeting did not compromise complex formation by Gorab. Gorab forms a homodimer upon Golgi localization, and it requires the integrity of its C-terminal coiled-coil domains for homodimerization (Fatalska et al., 2021). We therefore transiently co-transfected Gorab transgenes tagged with either Myc or GFP but lacking the HTA tag into cultured *Drosophila* cells. As expected, the GFP- and Myc-tagged forms of Gorab colocalized to the Golgi (Fig. 2). However, if the Myc-tagged Gorab was targeted to the mitochondria by an HTA tag, the majority of the co-expressed GFP-Gorab also relocalized to this compartment (Fig. 2). This indicates that the mitochondrially targeted Gorab can bring the non-mitochondrially targeted form to the mitochondria, strongly suggesting that their dimerization is not impaired by HTA-tag-mediated relocalization.

**Fig. 3.**
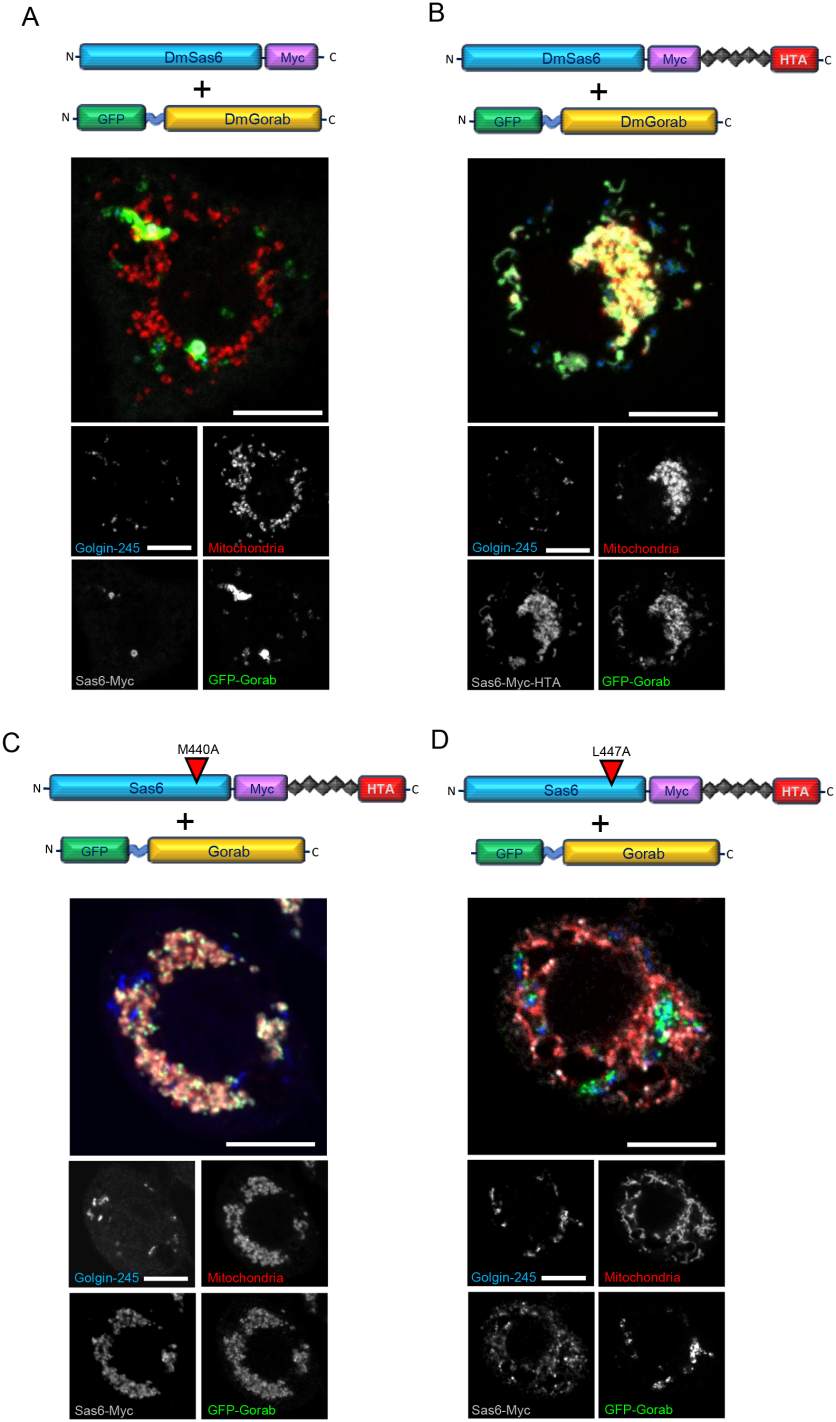
**Gorab distribution following expression of wild-type and mutant forms of mitochondrially relocalized Sas6.** (A-D) Cells were co-transfected with GFP-Gorab and either wild-type Sas6 lacking (A) or having (B) an HTA tag, or with HTA-tagged Sas6 carrying the M440A (C) or L447A (D) mutations. Cells were stained with MitoTracker Red, anti-Golgin245 antibody and anti-Myc antibody to reveal mitochondria, trans-Golgi and Sas6-Myc, respectively. Note that overexpression of HTA-tagged or untagged Sas6 and Gorab did not affect the centriole number in the transiently transfected cells (two centrioles/cell, *n*=80 transfected cells counted per construct). Scale bars: 5 μm. *n*=30 cells analyzed per construct transfected. Experiment repeated three times with the same result.

Knowing that complex formation was possible at the ectopic mitochondrial site, we asked whether targeting Sas6 to the mitochondria would result in the relocalization of Gorab away from the Golgi. We first transiently transfected cells with a construct to overexpress Sas6 lacking the HTA tag and found that this resulted in the formation of large cytoplasmic aggregates of Sas6 (Fig. 3A), similar to those reported in *Drosophila* spermatocytes upon Sas6 overexpression (Stevens et al., 2010). These Sas6 aggregates also displayed an elevated amount of co-expressed GFP-Gorab. The remaining GFP-Gorab signal was distributed evenly amongst the Golgi stacks (Fig. 3A). We also expressed wild-type Sas6, or its M440A and L447A mutant forms; Sas6 M440A binds Gorab with similar affinity to wild-type Sas6, whereas the L447A mutant binds Gorab 16 times more weakly (Fatalska et al., 2021). We found that all three forms of HTA-tagged Sas6 efficiently localized to the mitochondria and that the compact cytoplasmic aggregates observed in non-targeted Sas6 overexpression were no longer observed (Fig. 3B-D). Notably, both Sas6 wild type and Sas6 M440A recruited most of the co-expressed GFP-Gorab to the mitochondria and left only traces of GFP signal at the Golgi (Fig. 3B,C). By contrast, following overexpression of the mitochondrially targeted Sas6 L447A mutant, the majority of GFP-Gorab remained Golgi associated, in accord with its greatly reduced affinity for Gorab (Fig. 3D). Thus, exogenous Gorab can be relocalized from the Golgi onto Sas6 targeted to the mitochondrion through the previously mapped domain for the Sas6-Gorab interaction.

### Mitochondrially targeted Sas6 can recruit endogenous Gorab from the Golgi

**Fig. 4.**
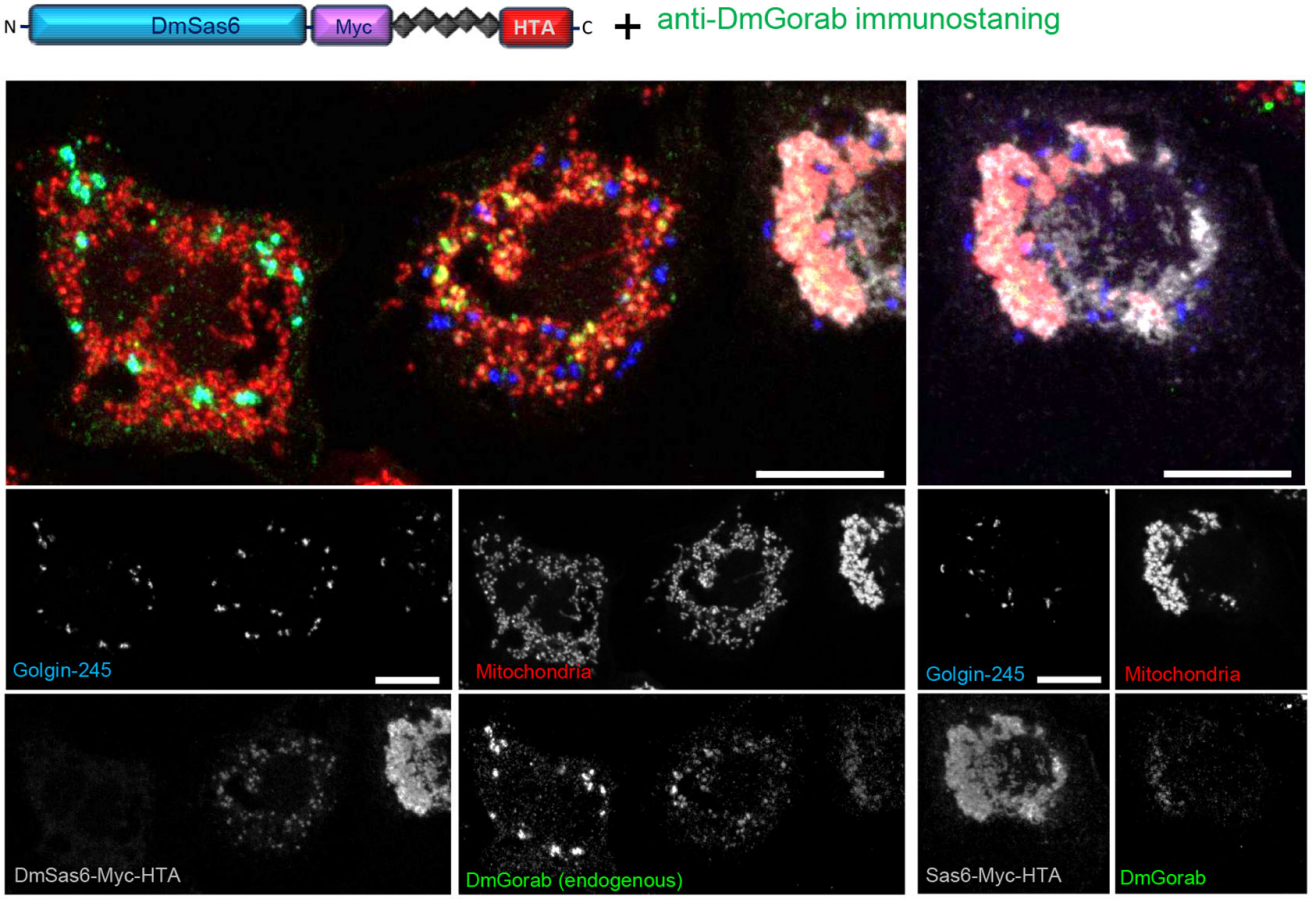
**Depletion of *Drosophila* Gorab Golgi pool by overexpression of mitochondrially targeted Sas6.** S2 cells were transiently transfected with Myc and HTA-tagged Sas6 construct and subsequently immunostained with anti-Gorab (green), anti-Myc (gray) and anti-Golgin245 (blue) antibodies. Cells not expressing (leftmost) and expressing (transfected, center and right) the Sas6-6xMyc-HTA construct are captured side by side in this field. The rightmost cell (also shown in the right panel in its entirety) has extremely high expression of the transgene, leading to aggregation of the mitochondria (stained by MitoTracker Red, in red). Scale bars: 5 μm. *n*=30 cells analyzed per construct transfected. Experiment repeated three times with the same result.

As the above experiments were performed in cells overexpressing exogenous Gorab, we considered it possible that the mitochondrially localized Sas6 was only absorbing the excess of Gorab. This led us to ask whether mitochondrially targeted Sas6 could also deplete endogenous Gorab from the Golgi. To test this, we transfected S2 cells with the HTA-tagged Sas6 construct alone and monitored Gorab levels by immunostaining with an anti-Gorab antibody (Kovacs et al., 2018). Following 24 h of the transient transfection, we could observe both cells expressing and not expressing the transgenes adjacent to each other in the dish (Fig. 4). Cells not expressing Sas6-Myc-HTA (negative for anti-Myc staining, leftmost cell in Fig. 4) had Gorab associated with the trans-Golgi marker. However, cells expressing Sas6-Myc-HTA were devoid of endogenous Gorab in the trans-Golgi structures (Fig. 4, middle and right cells). In these cells, the Gorab signal colocalized with Sas6-Myc-HTA and overlapped with a fraction of mitochondrial signal. This experiment demonstrated that the excess of Sas6 is able to capture endogenous Gorab and prevent it from localizing to the Golgi, in accord with the higher affinity of monomeric Gorab for Sas6 than for forming a homodimer for Golgi recruitment.

### Mitochondrially targeted *Drosophila* Sas6 can capture both *Drosophila* Gorab and human GORAB

**Fig. 5.**
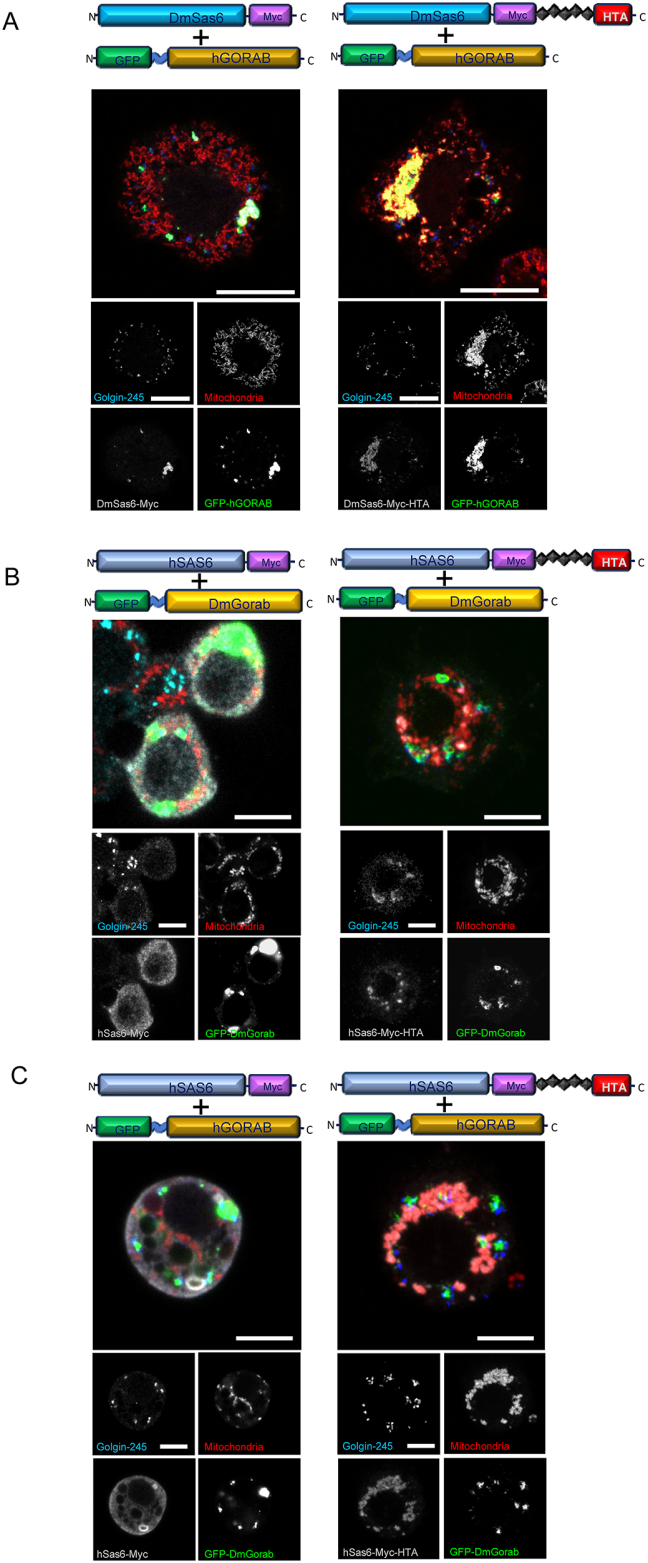
**Heterologous expression of human GORAB and SAS6 to test their interaction.** (A) *Drosophila* S2 cells transiently transfected with Myc- or Myc-HTA-tagged *Drosophila* Sas6 and GFP-tagged human GORAB. (B) *Drosophila* S2 cells transiently transfected with Myc- or Myc-HTA-tagged human SAS6 and GFP-tagged *Drosophila* Gorab. (C) *Drosophila* S2 cells transiently transfected with Myc- or Myc-HTA-tagged human SAS6 and GFP-tagged human GORAB. Cells are stained as indicated in the panels showing separated channels. Scale bars: 5 μm. *n*=30 cells analyzed per construct transfected. Experiment repeated three times with the same result.

The dual association of Gorab with the centriole and Golgi raises the question of whether its association and function at either or both these organelles was evolutionarily conserved. We approached this by asking whether *Drosophila* Sas6 could bind human GORAB. To test for such a heterologous interaction, we transiently transfected *Drosophila* Sas6, with or without a mitochondrial targeting sequence, and human GORAB into cultured *Drosophila* cells (Fig. 5). We found that transgenic human GFP-GORAB mainly localized to the trans-Golgi compartment in *Drosophila* S2 cells when co-expressed with *Drosophila* Sas6 not targeted to mitochondria (Fig. 5A, left panel). We also observed that, in this transgenic combination, a fraction of human GORAB colocalized with Sas6 aggregates, just as we observed with GFP-Gorab of *Drosophila* origin. However, when *Drosophila* Sas6 was targeted to mitochondria, the human GFP-GORAB signal colocalized with it (Fig. 5A, right panel). Thus, human GORAB appears to be able to complex with *Drosophila* Sas6. In contrast, we observed no colocalization when human SAS6 was co-expressed with either *Drosophila* Gorab or human GORAB even when human SAS6 was targeted to mitochondria by the HTA tag (Fig. 5B,C). As an alternative approach, we assessed the interaction of GST-tagged *Drosophila* Gorab or human GORAB with MBP-tagged *Drosophila* Sas6 or human SAS6 in an *in vitro* binding assay. This revealed that *Drosophila* Gorab could bind *Drosophila* Sas6 but not human SAS6. Human GORAB can bind *Drosophila* Sas6 but more weakly than *Drosophila* Gorab. The binding of human GORAB to human SAS6 was barely detectable (Fig. S1). Together, these experiments indicate that *Drosophila* Gorab and human GORAB show a strong conservation that permits both molecules to associate with the Golgi in *Drosophila* cells and form a physical complex with *Drosophila* Sas6. On the other hand, they point to sequence divergence of the Sas6 molecule between human and *Drosophila* in the region of the Gorab interaction site, such that *Drosophila* Sas6 is better able to bind the apparently more highly conserved Gorab molecule.

## DISCUSSION

Our previous study led us to hypothesize that the dynamic distribution of *Drosophila* Gorab between the centriole and Golgi depends upon its relative affinities for Sas6, required for localization to the centriole, and Rab6, required for Gorab to localize to the Golgi (Fatalska et al., 2021). We showed that the *Drosophila* Gorab monomer bound to Sas6 does not bind Rab6 but it binds Sas6 with a dissociation constant (Kd) of 47 nM. This compares to the Kd for the binding of the Gorab homodimer to Rab6 of 2.24 μM. This suggests that the equilibrium between Gorab monomers at the centriole and Gorab dimers at the Golgi can be determined by this 50-fold difference in relative binding efficiency to partners at the two sites. We chose to determine the consequences of this differential binding upon cytoplasmic localization by directing Sas6 to an ectopic site, the mitochondrion, using a 24-amino-acid motif from the C-terminal sequence from *Drosophila* Hid, an antagonist of Inhibitor of apoptosis protein (IAP). Expression of Sas6 tagged with this mitochondrial localization sequence in *Drosophila* cells targets Sas6 to mitochondria, where it is able to recruit not only exogenously provided Gorab, but is also able to deplete endogenous Gorab from the Golgi to the mitochondrion. These findings accord with the natural subcellular distribution of Gorab between the Golgi and centriole being set by the above relative binding affinities and the limiting amounts of Sas6 naturally present in the cell at the centriole.

**Fig. 6.**
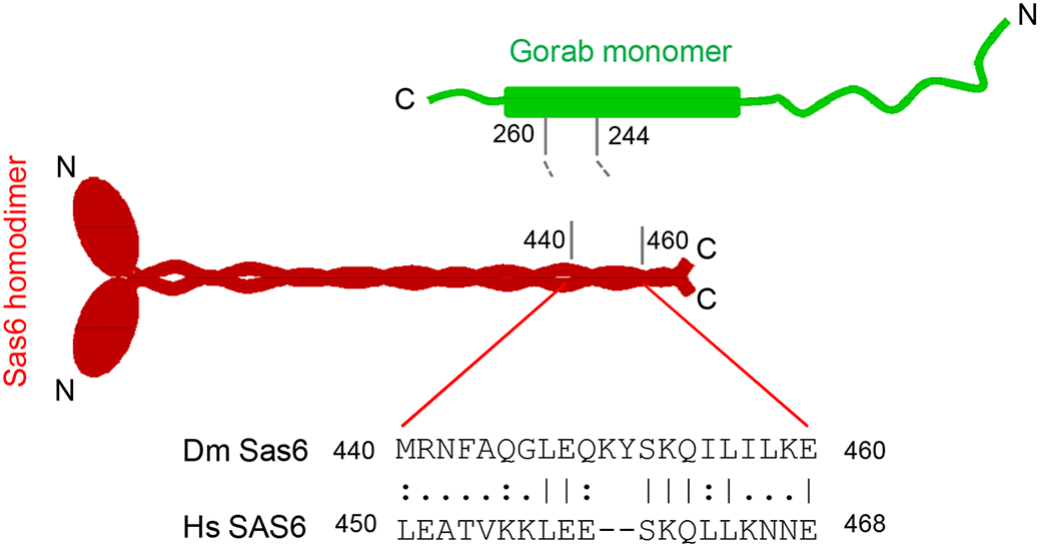
**Evolutionary divergence of Gorab-interacting motif in Sas6.** The indicated amino acid (aa)244-260 region of *Drosophila* Gorab monomer (green) interacts with the indicated C-terminal aa440-460 region of *Drosophila* Sas6 (red) homodimers (reproduced from Fatalska et al., 2021). The alignment of this 20-residue-long Gorab-interacting motif of *Drosophila* (Dm Sas6) and the corresponding region of the human (Hs SAS6) sequence is indicated. Numbers denote the amino acid positions in the protein sequences.

The Golgi is usually found in close association with the centrosome in interphase cells. Consequently, the differential affinity of Gorab for its Golgi-targeting and centriole-targeting partner may be crucial in regulating the correct balance of Gorab between its centrosomal and Golgi sites that are in close proximity. This would be of particular importance in *Drosophila* cells in which the physical association of Sas6 and Gorab is essential for centriole duplication (Kovacs et al., 2018; Fatalska et al., 2021). Mitochondrially targeted *Drosophila* Sas6 is able to bind and recruit both *Drosophila* Gorab and its human GORAB ortholog. By contrast, human SAS6 cannot bind fly Gorab, and its binding to human GORAB protein is barely detectable. This strongly suggests that the Gorab sequence has been more highly conserved, presumably through a need to dimerize and associate with the Golgi. *Drosophila* Sas6 makes its interactions with Gorab in part of the conserved coiled-coil domain to disrupt Gorab's dimerization. Sequence divergence between fly Sas6 and human SAS6 around the Gorab interaction site (Fig. 6) can account for the greatly diminished ability of human SAS6 to bind Gorab. This divergence of Sas6 perhaps reflects its differing requirements throughout the centriole duplication cycle in human and *Drosophila* cells; *Drosophila* Sas6 remains centriole associated throughout the duplication cycle whereas the Sas6 cartwheel is a transitory structure in human centriole duplication (Strnad et al., 2007; Guichard et al., 2010). In this light, we note that our previous study not only indicated the absolute requirement for Gorab for centriole duplication in *Drosophila* but also strongly suggested that GORAB participates in centriole duplication in cultured human cells (Kovacs et al., 2018). If this is indeed the case, then it is noteworthy that our previous immunostaining suggested that human GORAB remained associated with the centriole after Sas6 had been lost. This possibility of an alternative, Sas6-independent, pattern of association of human GORAB with the centriole in its duplication cycle requires further study.

## MATERIALS AND METHODS

### Plasmid generation

The C-terminal 24-amino-acid coding region containing the tail anchor of Hid was synthesized as a 111 bp-long sense-antisense oligonucleotide pair, which included a 3xGGGS coding sequence as a flexible linker upstream of HTA. The Gateway recombination cassette and a 6xMyc tag were amplified from the pAWM [*Drosophila* Genomics Research Center (DGRC) Stock 1104; https://dgrc.bio.indiana.edu//stock/1104; RRID:DGRC_1104] *Drosophila* expression vector. The pUb promoter and triple STOP codons together with the Hsp27 terminator sequence were amplified from the pUGW (DGRC Stock 1283; https://dgrc.bio.indiana.edu//stock/1283; RRID:DGRC_1283) *Drosophila* expression vector. The pUb promoter was subcloned into EcoRI-digested pUASTattB vector. The insertion of the pUb promoter left behind an EcoRI restriction site. The subsequent digest of pUAST-pUb with EcoRI was used as a backbone in a Gibson Assembly reaction (Gibson Assembly^®^ Kit, New England Biolabs). The Gateway cassette, 3xMyc, the HTA sequence, the 3xGGGS linker and the Hsp27 terminator sequence were incorporated in a one-step Gibson Assembly reaction into EcoRI-digested pUAST-pUb. Four clones were verified by sequencing, and the resulting Gateway-compatible destination vector was named as pUb-HTA. Gorab- and Sas6-coding sequences were cloned into pUb-HTA by an LR reaction, and the resulting clones were verified by sequencing. Although the white eye color marker and the 5xUAS sequences have no particular benefits in cultured cells, we decided to leave these sequences in the construct as this will enable the vector to be used in the generation of intact transgenic flies as well. The presence of the UAS element can further boost the expression of the relocalized proteins in a particular tissue if crossed to the appropriate GAL4-expressing line. Plasmids generated for these experiments are available from the authors upon request.

### *Drosophila* cell culture and immunostaining

*Drosophila* S2 cells (Gibco™, Thermo Fisher Scientific, #R69007) were cultured in 1× Schneider's *Drosophila* Medium (Gibco^TM^, Thermo Fisher Scientific, #21720024) supplemented with 10% fetal bovine serum (FBS) and 1:1000 Penicillin/Streptomycin. There were tests for mycoplasma contamination before the experiments. Expression vectors with or without the HTA were transiently transfected in a total amount of 1.2 μg onto 12-well tissue culture plates. Cells were incubated at 21°C for 48 h and then transferred onto Concanavalin A-coated coverslips in medium containing 1:4000 MitoTracker™ Red CMXRos (Thermo Fisher Scientific, #M46752) and incubated for 1 h. Coverslips were subsequently rinsed in PBS and fixed in 4% formaldehyde for 20 min. After fixation, cells were washed in PBS containing 0.1% Triton X-100 and blocked by incubation in 10% FBS for 1 h. After blocking, cells were incubated for 1 h at room temperature with the following primary antibodies: goat anti-Golgin245 (1:500; Riedel et al., 2016; Developmental Studies Hybridoma Bank, RRID:AB_2569587), mouse anti-cMyc 9E10 (1:800; Santa Cruz Biotechnology, #sc-40), guinea pig anti-Gorab (1:500; Kovacs et al., 2018). After three 10 min washes with PBS containing 0.1% Triton X-100, cells were incubated with appropriate secondary antibodies for 1 h at room temperature. After a final series of washes with PBS containing 0.1% Triton X-100, the coverslips were mounted in Vectashield.

### Microscopy and image analysis

Samples were imaged using a Leica Stellaris 8 confocal laser scanning microscope with a 63× objective. Recorded images were opened in ImageJ (https://imagej.nih.gov/ij/), and colocalization was first assessed by overlapping signals in merged color channels. The colocalization was further confirmed by RGB fluorescence intensity profile plots along a line drawn covering all four channels (as seen in Fig. 1D). For each sample, 30 cells expressing the constructs were analyzed. Cells expressing too high an amount of protein resulting in an oversaturated signal were excluded. Transient transfection experiments were repeated three times for each construct combination (biological replicates). No further statistical analyses were carried out as the relocalization was uniformly consistent in all cells investigated.

### Protein expression and *in vitro* binding assay

Recombinant proteins were expressed in *E. coli* strain BL21(DE3) following standard procedures. Briefly, bacteria were transformed with recombinant plasmids encoding the desired proteins and cultured at 37°C to an optical density of 0.5-0.7 (OD600) in Terrific Broth supplemented with appropriate antibiotics. Protein expression was induced with 0.5 mM isopropyl-b-D-1-thiogalactopyrano-side at 20°C overnight. Bacterial cells were harvested, resuspended in buffer A [20 mM Tris-HCl pH 7.5, 150 mM NaCl, 5% (v/v) glycerol, 1 mM dithiothreitol (DTT)], supplemented with EDTA-free protease inhibitor cocktail (Roche) and 0.1 mg/ml lysozyme (Sigma-Aldrich), and incubated on ice for 30 min. Cells were lysed by sonication and clarified by centrifugation at 15,000 ***g*** for 15 min at 4°C. *In vitro* binding assay was carried out by incubating the lysate containing bait GST-tagged protein on Glutathione-Sepharose 4B (GE Healthcare). After mixing by rotation for 1 h at 4°C, the beads were washed with buffer A [20 mM Tris-HCl pH 7.5, 250 mM NaCl, 5% (v/v) glycerol, 1 mM DTT, 0.5% (v/v) Triton]. Next, the prey MBP-tagged protein was added, and the mixture was incubated for 1 h at 4°C, followed by 3×10 min washes with buffer A. The proteins were eluted by boiling in Laemmli sample buffer and analyzed by SDS-PAGE with PageBlue protein staining (Thermo Fisher Scientific), followed by western blot analysis with appropriate antibodies.

## Supplementary Material

10.1242/biolopen.059545_sup1 Supplementary information
