# Supplementary material for: Targeting Drosophila Sas6 to mitochondria reveals its high affinity for Gorab
Source: Biol Open. 2022 Nov 18;11(11):bio059545. doi: 10.1242/bio.059545 (PMC9836085; doi:10.1242/bio.059545)
Supplement: Supplementary information [file biolopen-11-059545-s1.pdf]

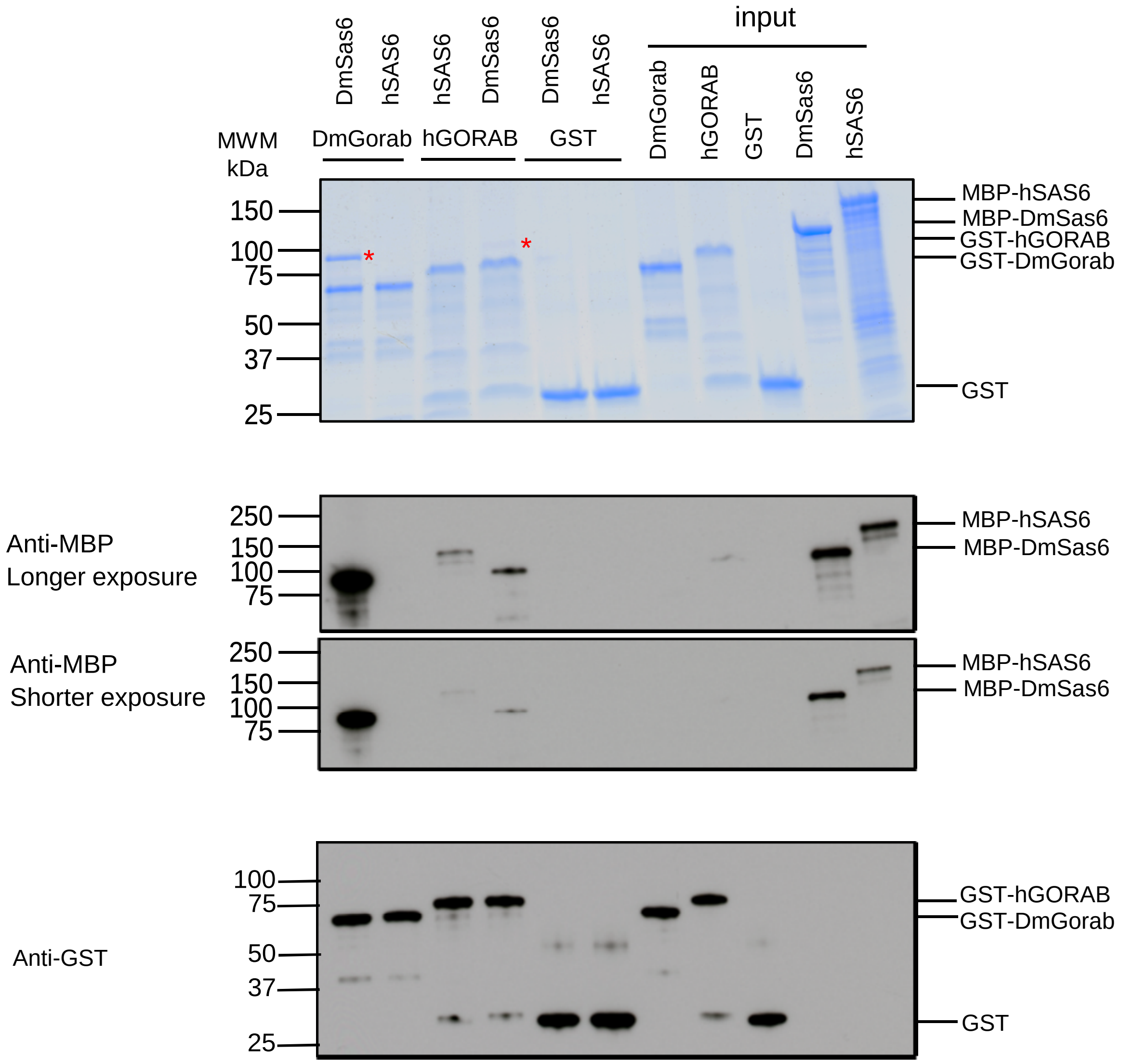

Fig. S1. Binding assay for Sas6 with Gorab from *Drosophila* and human origin. Upper panel: SDS-PAGE of the binding assay in which GST-DmGorab/hGORAB are the baits and MBP-DmSas6/MBP-hSAS6 are the preys. Middle panel: western blot showing MBP-DmSas6/MBP-hSAS6 pulled-down by Gorab and the inputs. Lower panel: western blot showing the GST-DmGorab/GST-hGORAB inputs.
